# A Longitudinal Investigation of Children’s Trauma Memory Characteristics and Their Relationship with Posttraumatic Stress Disorder Symptoms

**DOI:** 10.1007/s10802-021-00773-5

**Published:** 2021-02-03

**Authors:** Rosie McGuire, Rachel M. Hiller, Anke Ehlers, Pasco Fearon, Richard Meiser-Stedman, Sophie Leuteritz, Sarah L. Halligan

**Affiliations:** 1 Department of Psychology, University of Bath, Bath, UK; 2 Department of Experimental Psychology, University of Oxford, Oxford, UK; 3 Department of Clinical, Educational and Health Psychology, UCL, London, UK; 4 Department of Clinical Psychology, University of East Anglia, Norwich, UK; 5 Department of Psychiatry, University of Cape Town, Cape Town, South Africa

**Keywords:** Trauma, Memory, Narrative, Posttraumatic stress

## Abstract

While trauma memory characteristics are considered a core predictor of adult PTSD, the literature on child PTSD is limited and inconsistent. We investigated whether children’s trauma memory characteristics predict their posttraumatic stress symptoms (PTSS) at 1 month and 6 months post-trauma. We recruited 126 6–13 year olds who experienced a single-incident trauma that led to attendance at an emergency department. We assessed trauma memory disorganisation and sensory-emotional qualities through both narrative recall and self-report questionnaire, and PTSS at 1-month post-trauma and at 6-month follow-up. We found that, after controlling for age, children’s self-reported trauma memory characteristics were positively associated with their concurrent PTSS, and longitudinally predicted symptoms 6-months later. However, observable trauma memory characteristics coded from children’s narratives were not related to PTSS at any time. This suggests that children’s perceptions of their trauma memories are a more reliable predictor of the development and maintenance of PTSS than the nature of their trauma narrative, which has important implications for clinical practice.

## Introduction

After experiencing a single-incident trauma, many children will initially show signs of distress, with approximately 10–20% developing more chronic posttraumatic stress disorder (PTSD; Alisic et al., 2014; Hiller et al., 2016). Cognitive models of PTSD suggest the way an individual remembers a traumatic event (i.e., trauma memory quality) may be one key factor underlying the development and maintenance of elevated posttraumatic stress symptoms (PTSS; e.g., Brewin et al., 1996; Ehlers & Clark, 2000). Within the adult literature, there are some key characteristics of traumatic memories that are associated with PTSD development; disorganisation, sensory or perceptual qualities, and emotional content (Brewin, 2001; O’Kearney & Perrott, 2006). Less is known about trauma memory characteristics in relation to child PTSD. Given the significant developmental changes in memory processes from childhood to adulthood, it cannot be assumed that the same trauma memory characteristics would also predict the development and maintenance of child disorder (Nelson & Fivush, 2004; Peterson & Biggs, 1998).

The few studies that have investigated the association between trauma memory characteristics and PTSD in children have produced mixed results with contradictory conclusions (Kenardy et al., 2007; McKinnon et al., 2017; O’Kearney et al., 2007; Salmond et al., 2011). While O’Kearney et al. (2007) found that the narratives of children reporting a higher frequency of re-experiencing PTSS were more cohesive, less uncertain, and less sensory, other studies have found that children with higher levels of post-trauma distress have more disorganised narratives (Kenardy et al., 2007; Salmond et al., 2011). In their longitudinal investigation, McKinnon et al. (2017) found that lower levels of cohesion and more negative emotions in children’s trauma narratives were cross-sectionally associated with symptoms of acute stress disorder (ASD), although these qualities did not predict later PTSS (8–12 weeks post-trauma).

Some of these inconsistencies between studies may be explained by variation in the approach taken to code trauma narratives; however, even where the same coding manual was used, results have been contradictory (McKinnon et al., 2017; O’Kearney et al., 2007). Although participants in these studies were all recruited from emergency departments (EDs) following a single incident trauma, and were similar in age range (from 7–15 years to 8–17 years), there are still likely to be differences in trauma and participant characteristics across studies that may have contributed to discrepant findings. In addition, sample sizes have typically been modest, ranging from n = 50 (Salmond et al., 2011) to n = 87 (Kenardy et al., 2007), raising questions about power to detect effects reliably. Few studies so far have investigated self-reported trauma memory characteristics alongside coded trauma narratives. However, McKinnon et al. (2017) found self-reported sensory qualities to be a stronger predictor of post-trauma distress than the narrative characteristics. While narrative-derived measures of trauma memory quality have the advantage of being objectively rated, they are likely to be highly dependent on children’s verbal skills and what they choose to disclose. By contrast, self-reported memory characteristics may be less influenced by these factors but rely on children having sufficient understanding and insight to report reliably. Evidence of convergent findings based on these two measurement approaches is ideally needed.

Drawing on a longitudinal sample of young people exposed to single-incident traumas, the primary goal of this study was to examine the role of trauma memory qualities in relation to children’s acute and longer-term PTSS. To explore this, we conducted face-to-face interviews with 126 children, approximately 1-month following their exposure to a single incident trauma and subsequent attendance at an emergency department. At this assessment, young people completed a trauma narrative in which they described the index event, and this was coded for elements of disorganisation and sensory-emotional qualities. In addition, children completed self-report questionnaires assessing the quality of their trauma memories (disorganisation and sensory-emotional qualities) and their PTSS. PTSS were reassessed 6-months later. We examined associations between trauma memory qualities, using both narrative-derived and self-report measures, and PTSS at 1 month post-trauma and 6 months later. We controlled for key variables that may influence autobiographical memory and/or PTSS, namely age (Nelson & Fivush, 2004; Peterson & Biggs, 1998), sex (Fivush et al., 2000; Bokszczanin, 2007; Walker et al., 2004), and objective trauma severity (Foy et al., 1996; Trickey et al., 2012).

## Methods

### Participants

Participants were 126 6–13 year olds, recruited from UK EDs following exposure to acute trauma. Exclusion criteria were: the presence of a significant learning or neurodevelopmental disorder in the child that precluded mainstream schooling; injury resulting in a significant traumatic brain injury; and suspicion of intentional injury by the child (self-harm) or the parent (maltreatment). Research nurses initially identified 341 potentially eligible families, of whom 119 could not be contacted within 1-month, 65 were not interested or too busy, and seven believed assessments would be too distressing. Of the 151 families who initially agreed to participate, 19 could not complete the first study assessment within the required time frame. Consequently, the final sample for the longitudinal study was 132 children. One child declined to participate in the narrative task, and five were excluded from trauma memory assessments due to being unconscious during the traumatic event, based on a Glasgow Coma Scale score of below 13 upon arrival at the ED, indicating moderate loss of consciousness. Thus, the final sample for this study comprised 126 children, of whom 117 (93%) completed the 6-month follow-up assessment. There were no significant differences between those who agreed to participate and those who did not in age [*t* (330) = -1.37, *p* = 0.17], gender [*X*^*2*^ (1, *N* = 333) = 0.12, *p* = 0.73], or the presence of a head injury or concussion [*X*^*2*^ (1, *N* = 327) = 2.92, *p* = 0.09]. However, those who were admitted to hospital for observation postinjury were more likely to participate than those who were not [*X*^*2*^ (1, *N* = 305) = 6.58, *p* = 0.01].

## Procedure

 Full recruitment and procedural details are outlined in Hiller et al. (2018).

Researchers visited participants in their homes approximately one month after the traumatic event, then again 6 months later. At both home visits, participants completed a questionnaire-battery relating to the trauma and their mental health. At the 1-month assessment, children were also asked to give a detailed verbal narrative to the researcher of the traumatic event they had experienced. Narratives were audio recorded for later transcription. Participants completed the narrative task before the questionnaires were administered.

## Measures

### Descriptives

Child demographic and trauma-related information were obtained from ED notes and interviews with parents. The interview also asked parents whether they had any concerns about their child’s mental health prior to the trauma. Objective trauma severity was measured using the triage rating assigned to children by nurses upon arrival to hospital, which assesses the degree of injury to decide which patient requires treatment most urgently. This ranges from 1 = immediate care required, to 4 = less urgent.

### Child Posttraumatic Stress Symptoms

At both time points children completed the University of California at Los Angeles (UCLA) Posttraumatic Stress Disorder Reaction Index (PTSD-RI; Pynoos et al., 1998). The 17-item PTSS checklist covers DSM-IV-TR PTSD symptoms, with each item rated on a 0 (none of the time) to 4 (most of the time) scale. The measure provides a total PTSS severity score, ranging from 0 to 68 (α = 0.88), which was used as the primary outcome for the study.

### Child PTSD Diagnosis

Trained Researchers administered the Anxiety Disorder Interview Schedule- PTSD Module (ADIS-PTSD; Silverman et al., 1996), a well-validated diagnostic tool for PTSD based on DSM-IV-TR criteria, to parents and children. Diagnostic inter-rater agreement was established on the first 25% of the interviews (k = 1.00). Thereafter, approximately every sixth diagnostic interview was discussed at a consensus meeting to ensure consistency. Diagnostic information was used to characterise the sample. Additionally, we calculated symptom totals from the combined parent and child ADIS interviews and used the resultant scores in a sensitivity analysis to address the potential for single informant bias.

### Self-reported Trauma Memory Quality

At 1-month post-trauma, the Adapted Trauma Memory Quality Questionnaire (ATMQQ; Hiller et al., 2019) was administered to children, who rated agreement with 18-items on a scale from 1 (disagree a lot) to 4 (agree a lot). The ATMQQ includes all 11 items from the original Trauma Memory Quality Questionnaire (TMQQ; Meiser-Stedman et al., 2007a, b), indexing the sensory qualities of the child’s trauma memories and sense of nowness (e.g. “My memories of the frightening event are mostly pictures or images”). The original TMQQ has shown good internal consistency (Cronbach’s alpha of 0.82 in an emergency department sample) and has established construct validity, showing positive correlations with PTSD symptomatology and with re-experiencing symptoms in particular (Meiser-Stedman et al., 2007a, b). There is potentially conceptual overlap between TMQQ domains (sensory/intrusive memories) and re-experiencing symptoms. However, TMQQ scores have been found to predict unique variance in PTSS over and above re-experiencing symptoms alone (Meiser-Stedman et al., 2007a, b).

The ATMQQ, used in the current study, includes an additional seven item scale which measures trauma memory disorganisation (e.g. “I get mixed up about what order things happened during the frightening event”), adapted from the adult Trauma Memory Questionnaire (Halligan et al., 2003). In the current sample, internal consistency was strong for both the sensory qualities (α = 0.80) and disorganisation (α = 0.85) subscales, as well as the overall measure (α = 0.86). We also found that test–retest reliability for the ATMQQ was *r* = 0.61 for 1-month to 3-months (an additional data collection point not discussed in this paper, see Hiller et al., 2018 for details) and *r* = 0.59 for 3-months to 6-months.

### Trauma Narrative Characteristics

At 1-month post-trauma, participants were asked to provide a verbal narrative of the traumatic event they experienced. Participants were instructed to begin their narrative just before the event occurred and include whatever information they thought was important. The researcher did not interrupt the young person during the narrative. However, if they were struggling, basic prompts were used (e.g., *“and then what happened”?).*

**Narrative coding.** The narrative task was audio recorded and transcribed, and the resultant narrative was coded according to standard procedures (Foa et al., 1995; Halligan et al., 2003; Salmond et al., 2011; Van Minnen et al., 2002). Only codes from the trauma segment were used. This begins with the first expression of threat in the narrative and ends with the termination of immediate threat. This segment was divided into clauses or “chunks” that contained “only one thought, action or speech utterance”. These “chunks” were then coded as follows:

*Repetition.* Repeating a previous chunk with no new information (chunks clearly repeated purposefully for emphasis were not coded as repetition).

*Organised Thoughts.* Attempts to understand what is happening e.g. chunks indicating reasoning, realization, causal elaboration, hypothesis setting, decision-making, or planning. Use of words like ‘I remember’, ‘because’, ‘cos’ when trying to communicate their thinking.

*Disorganised Thoughts.* Involve/imply confused or disjointed thinking such as muddled thoughts, questioning, being overwhelmed, ambiguousness, expressions of uncertainty about the participant’s memory of events, etc. This code also includes non-consecutive thoughts, which jump around and lack temporal consistency.

*Negative Feelings*. Unpleasant emotions such as humiliation, fear, shock, and dissociative experiences such as freezing.

*Pain Utterances.* Utterances expressing pain experienced.

*Sensory Utterances.* Utterances referring to one of the five senses.

The scores for each coding category were transformed to z scores, to account for differences in verbosity between children. As per existing protocols, coding categories were then combined to create two overall variables of interest. The repetition, organised thoughts (reversed), and disorganised thoughts codes were combined to form the Elements of Disorganisation variable. The pain utterances, sensory utterances and negative feelings categories were combined to create the Sensory-Emotional Qualities variable.

**Global Coherence Rating of Narrative.** As well as using the first three coding categories to indicate disorganisation within the narrative (repetition, organised thoughts [reversed], disorganised thoughts), the global coherence of each narrative was also judged using a 10-point disorganisation rating scale (Halligan, et al., 2003), with 1 indicating a high level of organisation and coherence (Temporally sequential, high amounts of detail relevant to the event and/or reflective thinking) and 10 indicating a complete lack of memory for the event.

**Coding Reliability.** Coding was conducted by individuals trained in using the manual, and who were unaware of participant’s PTSD symptom severity. One individual coded all narratives and a second rater blind coded 20% of them. For all of the narrative coding categories, there was good agreement between raters (intraclass correlation coefficients [ICCs] = 0.73—0.94).

## Power Analysis

Study power was calculated in G*power (Faul & Erdfelder, 1992) based on the effect size for the association between parenting and child PTSS, the original key objective for this project, where we determined that a sample of 120 was required to detect an effect size of *r* = 0.25 (estimated longitudinal effect) with 80% power in a multiple regression with six predictors, at alpha = 0.05. Our study was not specifically powered based on PTSS-memory quality associations but such correlations reported in the extant literature have ranged from small (e.g., *r* = 0.24; O’Kearney et al., 2006) to large (e.g., *r* = 0.78; Meiser-Stedman et al., 2007a, b), which suggests that our sample size is adequate to examine equivalent effects.

## Data Analytic Plan

We conducted analyses in SPSS, with an alpha set at 0.05. As PTSS scores were positively skewed, we applied a square-root transformation to the dependent variables. Some skew remained, so associations were checked using Spearman’s Rho correlations, with any discrepancies noted. First, we ran bivariate correlations to investigate basic associations between the trauma memory narrative codes and self-report scores (1-month), and total PTSS (1-month and 6-months). We explored age, sex, and objective trauma severity (triage ratings) as potential covariates. The trauma memory measures comprised: the narrative coding categories (i) Elements of Disorganisation; (ii) Sensory-Emotional Qualities; the overall narrative Disorganisation Rating scale; and the self-reported disorganisation and sensory-qualities.

Next, we conducted a series of separate hierarchical linear regression analyses, exploring the role of 1-month memory characteristics in relation to 1-month and 6-month PTSS. We only included variables in these regressions where we found significant bivariate associations (*p* < 0.05). Finally, when exploring 1-month memory characteristics in relation to 6-month PTSS, we also conducted particularly stringent analyses that examined whether memory characteristics predicted later PTSS, even when controlling for initial symptom severity. That is, do memory characteristics longitudinally predict 6-month PTSS, even above initial symptom severity?

## Results

### Descriptive Statistics and Covariates

Key descriptives are in Table 1. Participants were 6–13 years old (*M* = 9.8, *SD* = 2.0). Sixty-two percent of the sample were boys. The majority of children had experienced a motor vehicle accident as their index trauma (51.6%). Other index traumas included serious accidental injury (30.1%; e.g. serious falls), an acute medical episode (7.1%; e.g. acute anaphylaxis), assault (2.4%), or other event (8.7%; e.g., house fire, near drowning). The largest proportion of children (44%) were given a triage rating of 1 on arrival at hospital, meaning they required immediate care; 23% were given a triage rating of 2 (very urgent); 20% had a triage rating of 3 (urgent); and 13% were rated 4 (less urgent). At 1-month post-trauma, 25.4% (n = 32) of children met criteria for PTSD, and 6 months later 9.5% (n = 12; data missing for 5 children) met diagnostic criteria. Participants’ narratives ranged from 12–521 chunks (*M* = 131.7*, SD* = 88.2), with the trauma segment ranging from 6–248 chunks (*M* = 58.4*, SD* = 43.2). Longer trauma segments were associated with higher PTSS at baseline (*r* = 0.19, *p* = 0.04), but not 6 months later (*r* = -0.02, *p* = 0.84). Child sex and triage were not significantly correlated with PTSS at either time, so were not included in further analyses (see Table 2 for details). Age was significantly negatively associated with 1-month and 6-month PTSS and was controlled for in the first step of all regressions.

**Table 1:** Descriptive Statistics for trauma memory measures and posttraumatic stress symptoms (PTSS)

	1 month		6 months	
Variables	*M*	*SD*	*M*	*SD*
Elements of Disorganisation	0.01	1.61	-	-
Sensory-Emotional Qualities	0.00	2.16	-	-
Disorganisation Rating Scale	4.76	2.38	-	-
ATMQQ- sensory qualities	22.21	6.52	-	-
ATMQQ- disorganisation	15.29	5.75	-	-
Total PTSS	18.40	13.24	12.93	12.00

Raw scores are shown here. Elements of Disorganisation and Sensory-Emotional qualities are calculated from z scores of coding categories.

*ATMQQ* Adapted trauma memory quality questionnaire

**Table 2:** Pearson’s and point-biserial bivariate correlations for all variables. Demographic/trauma factors are potential covariates, trauma memory measures are potential predictor variables and posttraumatic stress symptoms (PTSS) are the outcome variables for each regression

Variables	1	2	3	4	5	6	7	8	9
*Demographic/trauma factors*									
Age	-								
Sex	-0.05	-							
Triage	0.04	-0.00	-						
*Trauma memory measures*									
Elements of Disorganisation	0.07	0.14	0.07	-					
Sensory-Emotional Qualities	0.07	0.08	0.02	-0.06	-				
Disorganisation rating scale	-0.39***	-0.03	-0.08	0.34***	-0.26**	-			
ATMQQ- sensory	-0.21*	0.17	-0.04	-0.05	0.17	-0.10	-		
ATMQQ- disorganisation	-0.28**	0.02	-0.23*	0.21*	0.07	0.19	0.45***	-	
*PTSS*									
1 month	-0.20*	0.04	-0.06	-0.03	0.11	0.05	0.68***	0.55***	-
6 months	-0.23*	0.06	-0.04	-0.05	0.01	0.07	0.34***	0.38**	0.51***

*ATMQQ* Adapted Trauma Memory Quality Questionnaire.

*p < 0.05; **p < 0.01; ***p < 0.001

## Associations Between Trauma Memory Characteristics and PTSS

Table 2 presents correlations between trauma memory characteristics and PTSS. Both subscales from the ATMQQ were positively correlated with total PTSS at both time points, so were included in subsequent regression analyses. None of the trauma narrative indices were associated with child PTSS at either time point, so these were not examined further.

## Linear Regressions for 1-month Memory Characteristics Predicting 1-month PTSS

Linear regression analysis was conducted to examine ATMQQ subscales (disorganisation and sensory qualities) as concurrent predictors of child PTSS. Results are shown in Table 3. After controlling for age, trauma memory characteristics significantly predicted 48.5% of variance in 1-month PTSS, with both self-reported disorganisation and sensory qualities of memories independently associated with concurrent PTSS.

**Table 3:** Regression summaries of trauma memory variables predicting total posttraumatic stress symptoms (PTSS) at the 1 month and 6 months assessments, controlling for age in step 1

	∆*F*	*Df*	∆*R*^*2*^	β	
				ATMQQ- sensory	ATMQQ- disorganisation
1-month PTSS (UCLA PTSD-RI)	44.95***	(2, 89)	0.49	0.50***	0.35***
6-month PTSS (UCLA PTSD-RI)	8.13**	(2, 86)	0.15	0.25*	0.23*
1-month PTSS (ADIS)	19.08***	(2, 93)	0.28	0.40***	0.23*
6-month PTSS (ADIS)	7.16**	(2, 87)	0.13	0.26*	0.18

*ATMQQ* Adapted Trauma Memory Quality Questionnaire, *UCLA PTSD-RI* University of California at Los Angeles Posttraumatic Stress Disorder Reaction Index, *ADIS* Anxiety Disorder Interview Schedule

*p < 0.05; **p < 0.01; ***p < 0.001

## Linear Regressions for 1-month Memory Characteristics Predicting 6-month PTSS

Regression analyses were also conducted to investigate whether trauma memory characteristics at 1 month predicted PTSS 6 months later. After controlling for age, initial trauma memory characteristics predicted 15% of variance in 6-month PTSS, with both self-reported disorganisation and sensory qualities of memories as significant independent predictors (see Table 3). Next, this regression was rerun controlling for 1-month symptoms. After controlling for age and initial symptoms, the self-reported trauma memory characteristics of disorganisation and sensory qualities no longer predicted additional variance in 6-month PTSS.

## Sensitivity Analyses

We ran several additional analyses to probe the robustness of our findings. First, we re-ran regression analyses using expectation–maximisation to impute missing data. The pattern of results was the same, with ATMQQ sensory and disorganisation subscales each being associated with 1-month PTSS (sensory β = 0.54, *p* < 0.001; disorganisation β = 0.34, *p* < 0.001), and with 6-month PTSS (sensory β = 0.23, *p* = 0.01; disorganisation β = 0.21, *p* = 0.03), with similar effect sizes to those yielded by analyses of the complete dataset. This suggests that the missing data had little effect on our results.

Second, given the relatively wide age range of our sample and the significant change in cognitive abilities that occurs during this developmental period, we explored whether patterns of associations between the memory variables and PTSS duplicated in older (10–13 years) and younger children (6–9 years), based on a median split by age. When analyses were re-run within each age group, the pattern of findings was extremely similar to that reported for the whole sample. None of the narrative variables significantly correlated with PTSS at 1 month or 6 months, whereas in both younger and older children the ATMQQ subscales were associated with PTSS both cross-sectionally and longitudinally. The largest difference in magnitude of associations was for the prediction of 6-month PTSS by the ATMQQ disorganisation subscale where the association was statistically significant for older children (*r* = 0.40, *p* = 0.004) but not younger children (*r* = 0.30, *p* = 0.065). Overall, there was no clear evidence that the capacity of memory domains to predict child PTSS depends on child age.

Third, we also repeated the original analyses using the symptom total from the ADIS interview (based on combined parent–child report) as the outcome variable, instead of the UCLA-RI scores which are based on child self-report only. Again, the pattern of findings was broadly similar. The ATMQQ subscales, but not narrative scores, were positively correlated with PTSS at 1-month (sensory *r* = 0.51, *p* < 0.001; disorganisation *r* = 0.44, *p* < 0.001) and 6 months (sensory *r* = 0.33, *p* < 0.001; disorganisation *r* = 0.36, *p* < 0.001), and linear regression analyses also found ATMQQ scores to predict significant variance in PTSS as measured by the ADIS (see Table 3).

## Discussion

The aim of this research was to explore the role of children’s trauma memory characteristics in the acute aftermath of single-incident trauma exposure, both in relation to their acute and longer-term PTSS. We found only partial support for the theory that trauma memory characteristics were related to concurrent and longer-term outcomes. Self-reported trauma memory characteristics of disorganisation and sensory qualities at 1-month post-trauma were associated with 1-month and 6-month PTSS, but there were no equivalent associations for memory qualities based on children’s observed trauma memory narratives.

## Self-reported Memory Qualities

Our findings based on children’s self-reported memory characteristics provide support for existing cognitive models of PTSD, where trauma memories that contain more sensory/emotional components and have a weaker narrative structure are held to contribute to an individual’s ongoing sense of threat (e.g., Brewin et al., 1996; Ehlers & Clark, 2000). In the adult field, numerous longitudinal studies have reported that increased disorganisation and sensory qualities of the memory predict higher PTSS (e.g., Amir et al., 1998; Halligan et al., 2003; Murray et al., 2002). By contrast, trauma memory characteristics in children have been much less studied, longitudinal evidence is limited, and findings overall have been mixed (Kenardy et al., 2007; Meiser-Stedman et al., 2019; Salmond et al., 2011; McKinnon et al., 2017). We found that child self-reports of both sensory-emotional characteristics and disorganisation in trauma memories at 1-month post trauma are cross-sectionally related to levels of PTSS, and we replicated our findings using symptom scores based on clinical interviews as well as based only on child self-report.

We also found evidence of longitudinal associations between self-reported memory qualities at 1-month and PTSS at 6-month follow-up. However, these longitudinal associations were smaller in magnitude and did not remain after controlling for initial symptom severity. While this analysis is particularly conservative, it specifically examines whether initial trauma memory characteristics predict symptom change over time. Therefore, the lack of predictive effect of memory characteristics once 1-month symptoms are controlled suggests that memory qualities in the acute period do not drive the longer-term maintenance of PTSS beyond initial severity. It has been suggested that while trauma memory quality is likely to be a strong driver of initial post-trauma distress, negative cognitions and coping behaviours are important determinants of the maintenance of memory qualities and symptoms (Ehlers et al., 2004), and the current findings are consistent with that possibility. However, we also note that trauma memory characteristics are embedded within the PTSD diagnostic criteria. Given the lack of predictive effects for memory once initial symptoms are controlled for, we cannot rule out the possibility that PTSS-memory associations are a reflection of this overlap rather than evidence of a causal influence.

## Trauma Narrative Characteristics

In contrast to self-report findings, we found no evidence that memory qualities derived from children’s trauma narratives were associated with either concurrent acute PTSS or longer-term PTSS. Indeed, there was limited evidence that self-report ratings of memory quality were robustly correlated with narrative ratings, thus highlighting that the narratives and self-report may not tap into memory in the same way in children. Whilst the general consensus within the adult literature is that more disorganised trauma narratives are associated with higher levels of PTSS (e.g., Halligan et al., 2003; Harvey & Bryant, 1999; Jones et al., 2007), the evidence for the role of children’s trauma narrative characteristics is far less consistent.

There are several possible explanations for the current null findings based on analysis of child narratives. First, it is possible that narrative-based versus self-reported memory characteristics tap into fundamentally different processes. Whereas narrative scores may capture quality of memory recall, questionnaire scores examine self-perceptions of memory. However, it is possible that in children, where verbal expression may be limited by language development, children’s own perceptions of their memories give a more accurate picture than that provided by analysis of their verbal narratives. Notably, whereas in the adult literature there is evidence that narrative and self-report measures of trauma memory converge to some extent (e.g., Halligan et al., 2003), such convergence is lacking in the current study and in the broader child literature. Moreover, avoidance might mean that some children are reluctant to provide as full an account of their trauma as they can.

Second, almost all children in the current study experienced accidental injuries and their narrative accounts of these experiences were relatively brief. The brevity of children’s accounts may partly reflect their more limited linguistic and social competence relative to adults, but also the brief nature of the trauma itself for many. Whatever the underlying reasons, the fact that children in our study typically provided relatively short accounts of their trauma may have reduced the sensitivity of our narrative coding approach. Objective raters have to judge the disorganisation of the narrative on the basis of plausibility of the sequence of events reported. In addition, other studies have found that especially distressing moments that are later reexperienced may be omitted from narratives (Evans et al., 2007; Jelinek et al., 2010), without raters necessarily being able to spot the omission. Finally, the age range of participants in our study was younger than for any other study in the field. Instead of adapting existing approaches from the adult field, we may have needed to take a more developmentally informed perspective, for example by providing more structure for the trauma recall task (e.g., through a practice recall or additional prompting) or by adjusting our coding scheme to focus on narrative targets that may be more relevant in child populations.

## Clinical Implications

There could be various reasons for the lack of consensus between self-report and narrative coded memories and mental health, as we found here. A key consideration with self-report is the risk of single-informant bias inflating true associations, given young people were reporting on both their memory qualities and their symptoms (De Los Reyes et al., 2015). We attempted to address this by conducting a sensitivity analysis using PTSS scores from the ADIS clinical interview, which combines parent and child reports. This analysis yielded a similar pattern of results to those based on child report only. That said, coded memory qualities of a trauma narrative, particularly in lower-risk and child samples, may be less sensitive to capturing the individual’s true experience of recalling their trauma. This is reflected in a study by Knutsen & Jensen (2019), who found that whilst all young people receiving Trauma Focused Cognitive Behaviour Therapy (TF-CBT) develop a more organised narrative over the course of their treatment, there is no relationship between these narrative changes and PTSS. These findings, along with ours, imply that judging a child’s trauma narrative recall (e.g., as part of a clinical or risk assessment) may not be a useful marker for whether or not they may be experiencing or will go on to experience elevated PTSS. Rather, it is their perception or (mis)appraisal of their memories that seems more central, which could have important implications for clinical assessment and the focus of trauma-related interventions.

## Limitations and Future Research

While there are various strengths to this study, particularly the well-powered longitudinal design and use of both self-report and observation, the findings should also be considered in light of some limitations. First, it is important to note that the narrative task was always administered before the questionnaires, which means that we cannot rule out order effects. Although the pattern of associations between PTSS and narrative versus ATMQQ is markedly different and seems unlikely to be only due to administration order, future studies should introduce counterbalancing of assessments to address this concern. Second, we did not measure child verbal ability in the current study and whilst we controlled for age, verbal ability is likely to be a stronger correlate of a child’s capacity to give a coherent account of their trauma, as children of similar chronological ages can still vary widely in their narrative capacity. To investigate whether age-related limitations may have contributed a lack of predictive effects for narrative derived scores, we re-ran split half analyses by child age, and found that the pattern of memory-PTSS associations was similar for younger and older children in our sample. Nonetheless, future research on trauma narratives would benefit from the inclusion of measures of oral communication and verbal reasoning.

Finally, the demographics of the sample mean that findings cannot necessarily be generalised to other groups of trauma-exposed children. All children had experienced a single-incident, primarily accidental trauma, and we had some evidence that families of children with more severe injuries were more likely to participate in our study. We assessed child trauma history through questionnaire only in the current sample which had limited reliability (e.g., some families included multiple elements of the index event as different traumas, others did not include it at all), but based on this measure the majority of children had little or no prior trauma exposure. We also did not specifically ask about prior history of PTSD. The sample was predominantly Caucasian and participating required a parent to be willing and able to spare their time to be involved in the project, which likely skewed our sample towards parents who are keen to engage with their child about the trauma (Alisic et al., 2017; McGuire et al., 2019). Findings cannot necessarily be generalised to intentional trauma exposures such as child maltreatment, or to children living in more complex family environments, where prior experiences of trauma in the parent or the child may be important. Exploring the role of trauma memories in more varied samples remains an essential area of future research.

## Conclusion

We found that perceived trauma memory qualities in the weeks following children’s exposure to an acute trauma were associated with both their initial and longer-term PTSS. However, we found no robust evidence that memory qualities based on their narrative were associated with symptom severity. In contrast to the more consistent associations between memory recall and PTSS in the adult field (Halligan et al., 2003; Harvey & Bryant, 1999; Jones et al., 2007), our findings showed only partial support for the role of children’s trauma memories in driving PTSS in children exposed to single-incident trauma. Memory recall plays a central role in evidence-based treatments for child PTSD (i.e., trauma-focused CBT; National Institute for Health and Care Excellence, 2018), and our findings show the potential importance of this work not solely focusing on the content of the narrative but particularly on the perception children have around the nature of their memories.
